# Reorganising dermatology care: predictors of the substitution of secondary care with primary care

**DOI:** 10.1186/s12913-020-05368-2

**Published:** 2020-06-05

**Authors:** Esther H. A. van den Bogaart, Mariëlle E. A. L. Kroese, Marieke D. Spreeuwenberg, Herm Martens, Peter M. Steijlen, Dirk Ruwaard

**Affiliations:** 1 Department of Health Services Research, Care and Public Health Research Institute (CAPHRI), Faculty of Health Medicine and Life Sciences, Maastricht University, Duboisdomein 30, Maastricht, 6229 GT The Netherlands; 2 Research Centre for Technology in Care, Zuyd University of Applied Sciences, Heerlen, the Netherlands; 3 Department of Dermatology and GROW School for Oncology and Developmental Biology, Maastricht University Medical Centre+, Maastricht, The Netherlands

**Keywords:** Substitution, Referral decision, Dermatology, Primary care, Outpatient care

## Abstract

**Background:**

The substitution of healthcare is a way to control rising healthcare costs. The Primary Care Plus (PC+) intervention of the Dutch ‘Blue Care’ pioneer site aims to achieve this feat by facilitating consultations with medical specialists in the primary care setting. One of the specialties involved is dermatology. This study explores referral decisions following dermatology care in PC+ and the influence of predictive patient and consultation characteristics on this decision.

**Methods:**

This retrospective study used clinical data of patients who received dermatology care in PC+ between January 2015 and March 2017. The referral decision following PC+, (i.e., referral back to the general practitioner (GP) or referral to outpatient hospital care) was the primary outcome. Stepwise logistic regression modelling was used to describe variations in the referral decisions following PC+, with patient age and gender, number of PC+ consultations, patient diagnosis and treatment specialist as the predicting factors.

**Results:**

A total of 2952 patients visited PC+ for dermatology care. Of those patients with a registered referral, 80.2% (*N* = 2254) were referred back to the GP, and 19.8% (*N* = 558) were referred to outpatient hospital care. In the multivariable model, only the treating specialist and patient’s diagnosis independently influenced the referral decisions following PC+.

**Conclusion:**

The aim of PC+ is to reduce the number of referrals to outpatient hospital care. According to the results, the treating specialist and patient diagnosis influence referral decisions. Therefore, the results of this study can be used to discuss and improve specialist and patient profiles for PC+ to further optimise the effectiveness of the initiative.

## Background

Over the course of the last decade, global expenditure on healthcare as a share of world income has been increasing [1–3]. In the coming decades, healthcare spending is even expected to increase faster than prosperity [2, 4]. The population is ageing, and other explanations for rising expenses, such as technological development and lagging productivity, are likely to remain applicable in the future.

As a way to control costs and the utilisation of healthcare services, several countries (e.g., the Netherlands, the UK, Spain and Scandinavian countries) have implemented a gatekeeper system [5–8]. In these systems, general practitioners (GPs) fulfil an important role in patients’ further access to healthcare [5]. In addition, hospital care and specialist care (except emergency care) are accessible only upon referral from a GP. Since the literature shows that these systems lead to lower use of health services [9], more appropriate and more effective healthcare use [10] and lower expenditures [11], it is beneficial to further strengthen the position of primary care. Therefore, there have been many attempts to improve the effectiveness and efficiency of primary care and the referral process to outpatient hospital care to strengthen healthcare sustainability [12, 13].

Since 1972, healthcare expenditure as a percentage of the gross domestic product (GDP) has been increasing annually in the Netherlands [14]. Therefore, guaranteeing the financial sustainability of the healthcare system in the future is high on the Dutch political agenda [15].

To provide better care at lower costs, so-called pioneer sites have been appointed by the Minister of Health in the Netherlands [16]. At these pioneer sites, health insurers, care providers and patient organisations join forces to establish initiatives to improve the quality of care and reduce healthcare costs. The main goal of these initiatives is to accomplish the three dimensions of the Triple Aim principle proposed by Berwick et al. [17]. This principle focuses on reducing the per capita cost of healthcare, improving the health of the population and improving the patient experience of care. The ‘Blue Care’ pioneer site in the Maastricht-Heuvelland region has implemented several initiatives, one of which is Primary Care Plus (PC+). PC+ uses the concept of substitution, which focuses on shifting specialised care to less expensive and more accessible primary care [18]. The aim of PC+ is to achieve substitution by stimulating integrated care through the facilitating of consultations with medical specialists in the primary care setting. Internationally, comparable models of care are implemented, as for example specialist outreach services and shifted outpatient clinics [12, 13, 19, 20].

One of the specialties involved in PC+ is dermatology. Specialised dermatology care is in high demand due to the increase in the number of patients with dermatological complaints visiting their GPs [21, 22]. Skin conditions are among the most common diseases that are encountered by GPs and for which patients are referred to secondary care [21, 23, 24]. In the Netherlands, 14% of all GP consultations are related to a dermatological disorder [25]. In addition, the number of GP consultations for suspected lesions is increasing by 7.3% annually, and further increases are expected [26, 27]. Along with media campaigns aimed at increasing awareness about the danger of skin cancer and the ageing population [28], the increase in the number of dermatology-related consultations will lead to a growing demand for dermatology-related healthcare services. In addition, GPs often have a lack of dermatological knowledge, which is a reason for diagnostic uncertainty and the experience of difficulties with the diagnosis and treatment of skin disease [29–31]. Moreover, there is large variation in GP referrals to specialised medical care, which is caused by many factors, such as uncertainty about the diagnosis, perceived seriousness of the skin disease and patient preference [32, 33]. GPs’ referral decisions are crucial for the patients’ progress through the healthcare system and, moreover, for the costs of the healthcare system [34]. Therefore, with PC+, the use of specialist medical expertise in primary care can be strengthened and expanded and unnecessary referrals to (expensive) outpatient hospital care can be avoided.

Because of the novelty of PC+ at its initiation in 2014, clear guidelines for GPs about the exact type of patients and complaints to be referred to PC+ were lacking. Therefore, this study explores referral decisions following PC+ dermatology care and the influence of predictive patient and consultation characteristics. The results of this study could contribute to the development of patient profiles and input for the optimisation of the PC+ process.

## Methods

### Design

This retrospective study uses clinical data on referral decisions from patients who received dermatology care in PC+ from January 2015 to March 2017.

### Setting

PC+ is an initiative implemented in the pioneer site Blue Care, located in the Maastricht-Heuvelland region, in which 81 GPs in 55 GP practices care for a population of approximately 170,000 people [35]. In this region, different organisations work together and developed the PC+ intervention to substitute specialised medical care with primary care [36]. After a pilot, in which medical specialist performed consultations in GP practices, PC+ was implemented on a larger scale with two independent PC+ centres located in the city of Maastricht [37, 38]. This allowed GPs within the region to refer patients to a medical specialist in a neutral primary care setting, with GPs remaining responsible for their patients throughout the whole PC+ care process.

The focus of this study was on dermatology care in the current PC+ setting. Together with orthopaedics, internal medicine, neurology, otolaryngology, ophthalmology, and rheumatology, dermatology has been included in the two PC+ centres from the beginning. Over time, more medical specialties, including paediatrics, gynaecology, urology and a multidisciplinary back pain consultation facility with anaesthesiology and orthopaedics focusing on chronic pain, have been added. Between January 2015 and March 2017, 10,029 patients visited PC+. With 2952 patients, dermatology accounted for almost one-third of all patients in PC+. The distribution of patients among the different medical specialties is shown in Table 1. The low numbers of patients for some medical specialties were mainly caused by their later influx into PC+ and the lack of personnel for some specialties to organise PC+ consultations on a regular basis.

**Table 1:** Number of patients visiting Primary Care Plus for the different medical specialties (*N* = 10,029)

Medical specialty	Number of patients % (N)	Start in PC+
Dermatology	29.4% (2952)	January 2015
Orthopaedics	17.0% (1708)	January 2015
Internal medicine	2.9% (291)	January 2015
Neurology	6.4% (638)	January 2015
Otolaryngology	18.1% (1815)	January 2015
Ophthalmology	10.4% (1044)	January 2015
Rheumatology	5.6% (559)	January 2015
Paediatrics	0.5% (50)	November 2015
Gynaecology	6.6% (659)	December 2015
Urology	1.5% (149)	March 2016
Back pain consultation facility	1.6% (163)	November 2016

### Intervention

In the PC+ centres, patients with low-complex and non-acute health problems are seen by a medical specialist during a maximum of two consultations, after a referral from their GP. The two PC+ centres operate according to the same method; however, they differ from each other based on the number of consultation hours and the number of different medical specialties. Specialists in PC+ are senior staff specialists working as employees in Maastricht UMC+. The senior staff requirement is part of the specialist profile for PC+, which was established based on previous research [37]. Specialists are paid according to the standard hourly rate. The costs of the space used by the specialist in PC+ is part of the consultation fee. Furthermore, care in PC+ is claimed as primary care performance, through which it can be offered at a lower price compared to secondary care and consultations are not subjected to the patient’s deductible.

**Fig. 1:**
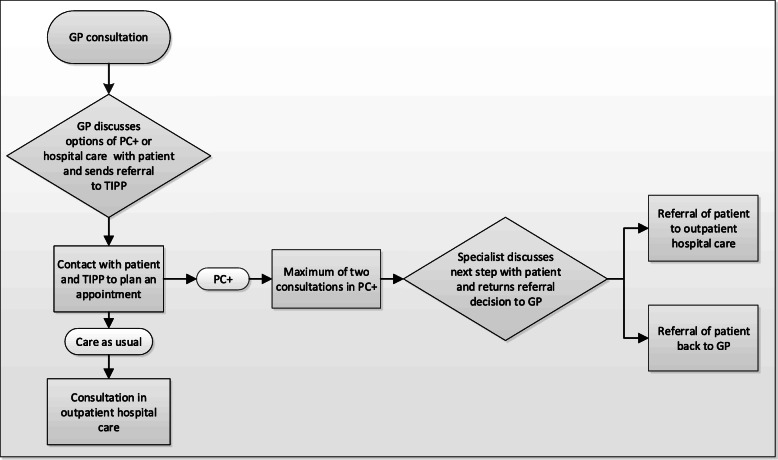
Flow chart of the Primary Care Plus process

The process of referring a patient to dermatology care in PC+ is similar to the process of referring a patient to outpatient hospital care and is shown in Fig. 1. GPs could refer a patient to PC+ when they had doubts about the diagnosis and/or treatment of patients with, what appeared to be low-complex and non-acute dermatology-related health problems. Profiles for patients eligible for PC+ were formulated by GPs and dermatologists during the study period and were made accessible online for GPs (see Additional file 1). These profiles were based on the experiences of GPs and medical specialists. In addition, it was assumed that patients referred to PC+ would have been referred to outpatient hospital care in a (hypothetical) situation in which PC+ was not available. The final decision to refer a patient to PC+ or to refer to care as usual (outpatient hospital care) was made based on consultation between the GP and the patient. After the decision was made, the referral was first sent to the Transmural Interactive Patient Platform (TIPP), which plans and registers referrals to medical specialists (either in PC+ or outpatient hospital care). In PC+, patients were seen by a dermatologist, and if necessary, dermatologists were able to perform cryotherapy, skin biopsies, blood tests, microbiology and Wood’s light investigation. Specialists treated patients and/or provided advice for GPs on further treatment strategies.

In this study, data from all patients visiting PC+ for dermatology care were collected.

### Outcome measures

The primary outcome in this study was the referral decision following PC+ (i.e., referral back to the GP or referral to outpatient hospital care). The independent variables were the consultation-related factors: number of PC+ consultations, treating specialist and patient diagnosis. The treating specialist was the specialist who treated the patient during the last PC+ consultation. In addition, the ‘treating specialist’ variable was divided into four categories: the first three categories included the three specialists who had performed the most PC+ consultations, and the fourth category included all other dermatologists working in PC+. The three specialists who had performed the most PC+ consultations had worked in PC+ since the beginning of the study period (January or February 2015). The specialists in the ‘other dermatologists’ category had started working in PC+ at some point during the study period (between January 2015 and October 2016). Patient diagnosis was defined as the diagnosis determined by the specialist during the last PC+ consultation according to the International Classification of Diseases (ICD-10) [39]. This variable was divided into mutually exclusive categories (meaning that patients could be placed in only one diagnosis category): the first ten categories included the ten most common diagnoses in PC+, an 11th category included all other diagnoses and a 12th category was for unknown diagnosis. The corresponding ICD-10 codes of the ten most common diagnoses in PC+ are presented in Additional file 2. In addition, patient age (in years) and gender were used.

### Statistical analysis

Continuous data are presented as the means and standard deviations (SDs). Categorical data are presented as the counts and percentages. Consultation-related factors and patient-related factors were compared between the two possible referral decisions following a PC+ consultation: (1) referral back to the GP or (2) referral to outpatient hospital care. An independent-samples t-test was used to compare the continuous data, and Pearson’s χ^2^ test was used to compare the categorical data. *P*-values ≤0.05 were considered statistically significant.

To describe variations in referral decisions, stepwise logistic regression modelling was used, with the decision to refer to outpatient hospital care as a binary yes/no variable. First, univariate logistic regression analysis was used to evaluate the relation between the primary outcome and the independent variables (predictors). Predictors with a *p*-value of ≤0.15 were included in the multivariable logistic regression analysis. For categorical variables, the variable was included when one or more categories had a *p*-value of ≤0.15. In this multivariable model, backwards elimination of the included variables was performed. The results were presented as unadjusted and adjusted odds ratios (ORs and AORs, respectively) with 95% confidence intervals (95% CIs), supplemented by the average marginal effects (AMEs). AMEs represent the difference in the adjusted predictions of the dependent variable relative to the reference group and improve the interpretability of the results [40]. With regard to the categorical variables treating specialist and diagnosis, the category within these variables that had an outpatient hospital care referral rate that was closest to the total average of that variable and that had a reasonable sample size was selected as the reference group. The explained variation in the regression model was measured by the Nagelkerke pseudo *R*^2^ [41].

Analyses were performed using SPSS software for Windows version 24.0 (SPSS Inc., Chicago, IL, USA) and R Studio (R Studio, Boston, MA).

## Results

Between January 2015 and March 2017, 2952 patients visited PC+ for dermatology care. The referral decision following PC+ was unknown for 140 patients; therefore, these patients were excluded from the analysis. These patients did not differ from the included patients in terms of age or gender (*p* = 0.748 and *p* = 0.430, respectively) (see Additional file 3). However, the excluded patients had significantly fewer PC+ consultations (*p* = 0.009). Furthermore, there was a difference in the distribution of treating specialists and diagnoses between the included and excluded patients (*p* = 0.002 and *p* ≤ 0.001, respectively).

The remaining 2812 patients had a total of 3355 PC+ consultations (average of 1.19, SD = 0.4 consultations). Following PC+, 80.2% (*N* = 2254) of the patients were referred back to their GPs, and 19.8% (*N* = 558) were referred to outpatient hospital care for further treatment/examination (see Table 2).

**Table 2:** Overview and comparisons of Primary Care Plus patients and consultation characteristics

	Total (N = 2812)	Referred back to GP 80.2% (N = 2254)	Referred to hospital care 19.8% (N = 558)	*p*-values
**Age in years (mean ± SD)**	47.7 ± 20.9	46.8 ± 21.0	51.5 ± 20.4	≤0.001**
**Gender – male % (N)**	41.2 (1159)	40.9 (923)	42.3 (236)	0.563
**Number of consultations (mean ± SD)**	1.19 ± 0.4	1.20 ± 0.4	1.16 ± 0.4	0.045*
**Specialist**				≤0.001**
Specialist 1% (N)	53.6 (1508)	84.6 (1276)	15.4 (232)	
Specialist 2% (N)	25.6 (721)	76.0 (548)	24.0 (173)	
Specialist 3% (N)	11.1 (311)	72.3 (225)	27.7 (86)	
Other % (N)	9.7 (272)	75.4 (205)	24.6 (67)	
**Diagnosis**				≤0.001**
Naevi % (N)	14.5 (407)	77.9 (317)	22.1 (90)	
Premalignant dermatosis % (N)	9.4 (264)	81.8 (216)	18.2 (48)	
Benign tumours % (N)	8.5 (238)	84.9 (202)	15.1 (36)	
Other eczema% (N)	7.8 (219)	93.2 (204)	6.8 (15)	
Acneiform dermatoses % (N)	6.1 (172)	87.2 (150)	12.8 (22)	
Inflammatory dermatoses % (N)	5.7 (161)	70.2 (113)	29.8 (48)	
Dermatoses due to microorganisms % (N)	5.3 (149)	93.3 (139)	6.7 (10)	
Malignant dermatoses % (N)	5.2 (146)	26.0 (38)	74.0 (108)	
Hair and nail disorders % (N)	3.7 (103)	95.1 (98)	4.9 (5)	
Pigment disorders % (N)	3.3 (94)	85.1 (80)	14.9 (14)	
Other % (N)	23.2 (653)	85.9 (561)	14.1 (92)	
Unknown % (N)	7.3 (206)	66.0 (136)	34.0 (70)	

* P < 0.05; ** P < 0.001

PC+ patients referred to outpatient hospital care were significantly older than those referred back to their GPs (*p* ≤ 0.001). There was no significant difference between the two groups with regard to gender (*p* = 0.563). PC+ patients referred to outpatient hospital care had significantly fewer PC+ consultations (*p* = 0.045). However, the difference was very small and therefore was not very clinically relevant. In addition, there were differences in the distribution of the referral decision by treating specialist and diagnosis within PC+ (both *p* ≤ 0.001).

### Specialists

In total, 12 different dermatologists worked in PC+ during the study period. However, the number of PC+ consultations held by these specialists varied greatly. There were three specialists who had seen the vast majority of patients. These three specialists saw approximately 90% (*N* = 2540) of the patients visiting PC+ for dermatology care during the study period.

### Predictors of a referral to outpatient hospital care

The results of the univariate and multivariable logistic regression analyses are shown in Table 3. Variables with a *p*-value ≤0.15 in the univariate analysis (age, number of consultations, treating specialist and diagnosis) were included in the multivariable logistic regression analysis. In the multivariable model only, treating specialist and patient diagnosis were retained as variable that independently influenced a referral to outpatient hospital care.

**Table 3:** Logistic regression analysis of referral to outpatient hospital care among dermatology patients in Primary Care Plus (*N* = 2812)

Predictors	Univariable model			Final model		
	Unadjusted OR (95% CI)	AME	***p***-value	Adjusted OR (95% CI)	AME	***p***-value
**Age†**	1.12 (1.07–1.17)	0.02	≤0.001***	…^a^		
**Gender - male**	1.06 (0.88–1.28)	0.01	0.510	…		
**Number of consultations**	0.79 (0.62–1.00)	− 0.04	0.054	…^a^		
**Specialist**						
Specialist 1	0.55 (0.45–0.66)	−0.10	≤0.001***	…^b^		
Specialist 2	1.40 (1.14–1.72)	0.06	≤0.001***	1.88 (1.48–2.39)	0.09	≤0.001***
Specialist 3	1.64 (1.26–2.15)	0.09	≤0.001***	1.97 (1.44–2.69)	0.09	≤0.001***
Other specialists	1.36 (1.02–1.83)	0.05	0.038*	1.80 (1.29–2.52)	0.08	≤0.001***
**Diagnosis**						
Naevi	1.18 (0.91–1.52)	0.03	0.215	1.30 (0.88–1.92)	0.04	0.195
Premalignant dermatosis	0.89 (0.64–1.23)	−0.02	0.477	…^b^		
Benign tumours	0.70 (0.49–1.01)	− 0.05	0.058*	0.83 (0.51–1.33)	−0.03	0.433
Other eczema	0.28 (0.16–0.47)	−0.14	≤0.001***	0.36 (0.19–0.66)	−0.11	≤0.001***
Acneiform dermatoses	0.58 (0.36–0.91)	−0.08	0.018*	0.66 (0.38–1.14)	−0.05	0.136
Inflammatory dermatoses	1.78 (1.26–2.53)	0.11	≤0.001***	2.12 (1.33–3.38)	0.14	0.002**
Dermatoses due to microorganisms	0.28 (0.15–0.53)	−0.14	≤0.001***	0.32 (0.16–0.66)	−0.11	0.002**
Malignant dermatoses	14.00 (9.54–20.53)	0.57	≤0.001***	12.98 (7.96–21.17)	0.55	≤0.001***
Pigment disorders	0.70 (0.39–1.24)	−0.05	0.223	0.77 (0.40–1.49)	−0.03	0.441
Hair and nail disorders	0.20 (0.09–0.49)	−0.16	≤0.001***	0.23 (0.09–0.59)	−0.13	0.002**
Other diagnosis	0.60 (0.47–0.76)	−0.07	≤0.001***	0.71 (0.49–1.05)	−0.04	0.086
Unknown diagnosis	2.23 (1.65–3.03)	0.15	≤0.001***	2.24 (1.45–3.45)	0.15	≤0.001***

*Note: OR* Odds ratio*, Cl* Confidence interval*, AME* Average marginal effects

* P ≤ 0.15; ** P < 0.01; *** P < 0.001***†****Age was rescaled such that one unit is equal to 10 years*

^a^Variable not significant in final model

^b^Reference category for the adjusted OR analysis

Patients treated by specialist 2 (AOR 1.88, 95% CI = 1.48–2.39, AME = 0.09), specialist 3 (AOR 1.97, 95% CI = 1.44–2.69, AME = 0.09) or another (less common) specialist (AOR 1.80, 95% CI = 1.29–2.52, AME = 0.08) were more likely to be referred to outpatient hospital care following PC+ than patients treated by specialist 1 (reference group). In addition, patients diagnosed with malignant dermatosis (AOR 12.98, 95% CI = 7.96–21.17, AME = 0.55) or inflammatory dermatoses (AOR 2.12, 95% CI = 1.33–3.38, AME = 0.14) and patients for whom the diagnosis was unknown (AOR 2.24, 95% CI = 1.45–3.45, AME = 0.15) were more likely to be referred to outpatient hospital care than patients diagnosed with premalignant dermatosis (reference group). On the other hand, patients diagnosed with other eczema (AOR 0.36, 95% CI = 0.19–0.66, AME = − 0.11), dermatoses due to microorganisms (AOR 0.32, 95% CI = 0.16–0.66, AME = − 0.11) and hair and nail disorders (AOR 0.23, 95% CI = 0.09–0.59, AME = − 0.13) were less likely to be referred to outpatient hospital care following PC+ consultations. The final model explained 19.3% of the variation in PC+ referral decisions (Nagelkerke *R*^2^ = 0.193).

## Discussion

This study explored referral decisions following dermatology care in PC+ and the influence of predictive patient and consultation characteristics on this decision. The results showed that the majority of the patients (80.2%) were referred back to their GPs following a consultation for dermatology care in PC+. This finding is in line with previous research suggesting that initiatives like PC+ have the ability to reduce outpatient hospital care referrals and/or increase the appropriateness of referrals made [13]. However, it is important to verify whether the assumption based on previous research that all patients would have been referred to secondary care if PC+ had not been available is also valid in this case [38].

Furthermore, the results showed that the treating specialist and patient diagnosis independently influenced referral decisions following dermatology care in PC+. Regarding the treating specialist, previous research by van Hoof et al. [37] indicated a profile for appropriate specialists in PC+. According to this profile, specialists should, in addition to having a certain degree of seniority, work according to a generalist approach and have an attitude that is consistent with the model of substitution. The extent to which the included specialists met this profile was not part of this study. However, the results indicated that the likelihood of patients being referred to outpatient hospital care was influenced by the treating specialists. A reason for this could be that the ability to work in a PC+ setting differs among specialists, and for example, specialists with a less generalist approach may refer patients to outpatient hospital care more often. Therefore, more research is needed to study the ability of specialists to work in PC+.

Regarding the patient diagnosis, the results provide an indication of diagnoses that are suitable for PC+. However, high referral rates to outpatient hospital care do not necessarily indicate that complaints leading to these diagnoses are inappropriate for PC+. For example, regarding malignant dermatoses, PC+ can function as a screening tool to prevent patients with an unjustified suspicion from being referred to hospital care for unnecessary testing and treatment. In addition, PC+ can improve early detection, and patients with more suspicious symptoms can be referred to secondary care for treatment, which may reduce mortality and improve quality of life [42]. In additions, diagnoses such as other eczema, dermatoses due to microorganisms and hair and nail disorders, which have low referral rates to outpatient hospital care, seem particularly suitable for PC+. Nevertheless, these diagnoses will not necessarily always be appropriate for PC+. GPs may also experience a (too) low threshold when referring patients to PC+ [37].

As suggested by van Hoof et al. [37], GPs and specialists should discuss appropriate and inappropriate complaints, symptoms and diseases for PC+. The results of this study can provide input for this discussion and can be used to further develop patient profiles for PC+ (see Additional file 1) if necessary. In addition, when a patient profile for PC+ is composed, appropriate and inappropriate diagnoses should be translated into the International Classification of Primary Care (ICPC) codes [43]. The ICPC-codes are used by every Dutch GP and function to categorise patients’ complaints, symptoms and diseases. One specific ICPC-code could ultimately lead to several diagnoses. In this study, the ICPC-codes were not available. Therefore, the patient diagnosis made by the specialist in PC+ was used as a predictor of the referral decision. Furthermore, the clear provision of advice from specialists to GPs when specialists refer patients back to GPs could contribute to a learning effect among GPs regarding the diagnosis or treatment of dermatology patients and whether to refer to PC+ or outpatient hospital care [44]. This feedback could contribute to bridging the knowledge gap between primary and secondary care [29].

The variation explained by the final model in this study was 19.3%, which implies that a lot of variation is explained by other (party unknown) variables that were not included in the model. According to the literature on GP referrals to hospital care, case-specific factors, such as the nature of the disease and the observed severity, influence the patient referrals [45]. In addition, other patient-associated factors than age and sex, such as the overall health status, insurance coverage, social class, needs and values, pressure for referral and preferences, may influence the referral decision [32, 33, 46].

The results showed that 29.4% of all patients visiting PC+ during the study period had dermatological complaints. This percentage is higher than the 14% of all Dutch patients visiting their GP with a dermatological complaint. However, it is difficult to compare these percentages, since not all medical specialties are represented in PC+. In addition, the medical specialties in PC+ did not have an equal number of consultation hours during the study period due to an unequal influx of patients and a lack of personnel for some medical specialties. Finally, PC+ is focused on a select group of patients with low-complex and non-acute dermatology related health problems who are eligible for this care (see Additional file 1).

Moreover, since PC+ focuses on non-acute and low-complex care, it is assumed that more serious diagnoses, such as malignancies, are not made more often in PC+ than in outpatient hospital care. Epidemiological data on dermatological conditions in primary care and hospital care in the Netherlands [47] and the data for patients diagnosed with malignant dermatoses in the present study are consistent with this observation. In PC+, 5.2% of the patients were diagnosed with malignant dermatoses, compared to 12% of patients in hospital care [47] (see Additional file 4). On the other hand, it is assumed that less serious diagnoses are made more often in PC+ than in outpatient hospital care, since the aim of PC+ is to substitute secondary care with primary care for low-complex care. Based on epidemiological data and the data in the present study, it can be concluded that this is the case for diagnoses such as naevi, benign tumours and inflammatory dermatoses.

In addition, there are various other approaches to reduce outpatient hospital care referrals and/or increase the appropriateness of referrals [13], such as the concept of teledermatology [22, 48] and the employment of GPs with special interests and the implementation of nurse-led services in these kind of settings [23, 49, 50]. Even though these initiatives show generally positive findings in terms of accessibility, waiting time and patient satisfaction [22, 51, 52], researchers also have critiqued the diagnostic accuracy of telemedicine [53], the lack of specific research on patient safety [54], and the limited evidence regarding cost-effectiveness [55].

### Limitations

The use of monitoring data limited the amount of information, and therefore predictors, for this study. Extending the data, for example with data from GP practices, can generate more useful information. Examples include the ICPC codes and registration of the severity of the complaints. By expanding the data, the referral decision may be better predicted and more and better information can be given back to GPs and medical specialists in order to improve the efficiency of PC+. In this study, data expansion was not possible because data from GP practices in this region are registered through different systems, which makes data linking complicated.

Additionally, a limited number of specialists were included in the present study. However, differences in the referral decisions of these specialists were observed. It was not possible to include more characteristics of the PC+ specialists in the present study since these variables may affect the anonymity of the specialists involved. However, the results of this study can be used as input for further research. Including more specialists and more characteristics in further research, such as specialist age and work experience, could contribute to more insight into the variation in specialists’ referrals and, therefore, to more input for PC+ the specialist profiles.

Furthermore, follow-up data of patients visiting PC+ for dermatology care were not taken into account. It is possible that patients who were initially referred back to their GPs had follow-up visits for the initial complaint in secondary care shortly having a PC+ consultation. If this pattern were to occur on a significant scale, PC+ would be less appropriate. Therefore, hospital data should be analysed. It is also relevant to determine whether the substitution effect is present in outpatient hospital care.

Finally, the referral decisions following the PC+ consultations of 140 patients and the diagnoses of 206 patients were missing. The results showed that patients excluded from the analysis differed from the included patients in terms of the number of PC+ consultations, and the distribution of treating specialists and diagnoses; therefore, selection bias may exist (see Additional file 3). However, only 140 of the 2952 patients needed to be excluded, which is a relatively low number. Furthermore, incomplete patient cases were partly caused by specialists becoming accustomed to the registration method at the beginning of PC+. Therefore, the degree of selection bias seems limited and it is not expected that the results were considerably influenced.

## Conclusion

To conclude, through the referral of a large number of patients back to their GPs following dermatology care in PC+, the number of referrals to hospital care can be limited; thus, dermatology care seems to be suitable for PC+. Both the treating specialist and the patient diagnosis influenced the referral decision. Therefore, the results of this study can be used to discuss and improve profiles for specialists and patients in PC+ to further optimise the effectiveness of the initiative. Besides insight into the influence on quality of care, further research is needed into the costs and volumes of dermatology care, both in PC+ and secondary care to determine if substitution of dermatology care actually occurs and healthcare costs are reduced.

## Supplementary information

**Additional file 1.** Patient profiles Primary Care Plus. Description of which dermatology complaints/care is appropriate to be referred to Primary Care Plus.

**Additional file 2.** Top ten diagnoses and ICD-10 codes. Top ten dermatology diagnoses in Primary Care Plus with corresponding ICD-10 codes.

**Additional file 3.** Comparison of patient categories. Comparison of patients and consultation characteristics of included and excluded patients.

**Additional file 4.** Distribution of dermatological complaints. Comparison of the distribution of dermatological complaints in primary care, Primary Care Plus and secondary care.

## Data Availability

The dataset used and analysed during the current study is available from the corresponding author on reasonable request.

## Abbreviations

AME: Average Marginal Effect
AOR: Adjusted odds ratio
CI: Confidence interval
GDP: Gross domestic product
GP: General Practitioner
ICD-10: International Classification of Diseases
ICPC: International Classification of Primary Care
Maastricht UMC+: Maastricht University Medical Centre+
OR: Odds ratio
PC+: Primary Care Plus
SD: Standard deviation
TIPP: Transmural Interactive Patient Platform
