# Supplementary material for: Reorganising dermatology care: predictors of the substitution of secondary care with primary care
Source: BMC Health Serv Res. 2020 Jun 5;20:510. doi: 10.1186/s12913-020-05368-2 (PMC7275501; doi:10.1186/s12913-020-05368-2)
Supplement: Supplementary file 1 — Additional file 1. Patient profiles Primary Care Plus. Description of which dermatology complaints/care is appropriate to be referred to Primary Care Plus. [file 12913_2020_5368_MOESM1_ESM.pdf]

Dermatology patient profile of PC+

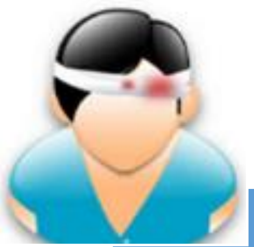

appropriate

appropriate

All care that does not need hospital care and when in doubt, for example:

- Doubt about malignancy
- All general dermatology as well as biopsies and nitrogen treatment
- One demand for care!

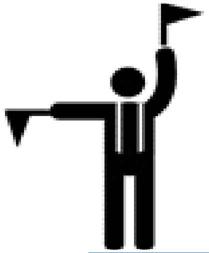

inappropriate

inappropriate

- Evident treatment trajectory in hospital (day treatment / OK / laser therapy)
- Acute care
- Excisions
- Phlebology (varicose veins)
- Open wounds
- Second opinion
- Biopsies in the face
- Genital Warts
- Oedema
