# Supplementary material for: Reorganising dermatology care: predictors of the substitution of secondary care with primary care
Source: BMC Health Serv Res. 2020 Jun 5;20:510. doi: 10.1186/s12913-020-05368-2 (PMC7275501; doi:10.1186/s12913-020-05368-2)
Supplement: Supplementary file 2 — Additional file 2. Top ten diagnoses and ICD-10 codes. Top ten dermatology diagnoses in Primary Care Plus with corresponding ICD-10 codes. [file 12913_2020_5368_MOESM2_ESM.docx]

**Diagnosis and corresponding ICD-10 codes**

Table 1 Top ten diagnoses in PC+ with corresponding ICD-10 codes

| **Naevi** |  |  |  |  |  |  |  |  |
| --- | --- | --- | --- | --- | --- | --- | --- | --- |
| D22.3 | D22.90 | D22.916 | D22.96 | D23.9 | L81.46 | Q82.222 | Q82.56 | Q82.810 |
| D22.312 | D22.91 | D22.92 | D22.97 | D48.5 | L81.47 | Q82.3 | Q82.57 | Q82.811 |
| D22.512 | D22.911 | D22.93 | D22.971 | L81.41 | L81.48 | Q82.53 | Q82.58 | Q82.813 |
| D22.513 | D22.914 | D22.94 | D22.98 | L81.410 | Q82.2 | Q82.54 | Q82.59 | Q82.9 |
| D22.9 | D22.915 | D22.95 | D22.99 | L81.45 | Q82.21 | Q82.55 | Q82.591 |  |
| **Premaligne dermatosis** | | |  |  |  |  |  |  |
| A63.8 | D03.9 | D07.4 | D47.0 | L44.82 | L57.1 | L90.0 | L90.02 | N89.4 |
| C90.2 | D04.9 | D29.9 | D48.1 | L57.0 | L85.85 | L90.01 | N48.0 | N90.4 |
| **Benign tumours** | |  |  |  |  |  |  |  |
| D17.9 | D21.92 | D23.91 | D23.917 | D23.923 | D23.930 | D23.98 | D76.3 | L72.1 |
| D18.1 | D21.93 | D23.911 | D23.918 | D23.924 | D23.931 | D23.99 | D76.31 | L72.2 |
| D21.14 | D23.0 | D23.913 | D23.92 | D23.927 | D23.95 | D24.0 | K13.71 | L72.8 |
| D21.5 | D23.24 | D23.914 | D23.920 | D23.928 | D23.96 | D36.1 | L72.0 | L72.81 |
| D21.9 | D23.33 | D23.915 | D23.921 | D23.929 | D23.97 | D36.11 | L72.01 | L72.9 |
| D21.91 | D23.90 | D23.916 | D23.922 | D23.93 |  |  |  |  |
| **Other eczema** | |  |  |  |  |  |  |  |
| I83.1 | L30.12 | L30.5 | L30.84 | L30.87 | L55.9 | L56.4 | L56.81 | L56.91 |
| L30.0 | L30.13 | L30.8 | L30.85 | L30.88 | L56.0 | L56.41 | L56.82 | L56.92 |
| L30.1 | L30.4 | L30.82 | L30.86 | L30.91 | L56.1 | L56.8 | L56.9 | L85.31 |
| L30.11 |  |  |  |  |  |  |  |  |
| **Acneiform dermatoses** | | |  |  |  |  |  |  |
| L70.0 | L70.05 | L70.41 | L70.83 | L71.0 | L71.81 | L71.9 | L73.1 | L73.82 |
| L70.01 | L70.1 | L70.5 | L70.84 | L71.01 | L71.82 | L71.91 | L73.2 | L73.83 |
| L70.02 | L70.2 | L70.8 | L70.85 | L71.02 | L71.83 | L72.82 | L73.8 | L73.84 |
| L70.03 | L70.3 | L70.81 | L70.86 | L71.1 | L71.84 | L73.0 | L73.81 | L73.9 |
| L70.04 | L70.4 | L70.82 | L70.9 | L71.8 |  |  |  |  |
| **Inflammatory dermatoses** | | | |  |  |  |  |  |
| D69.0 | H60.9 | L12.1 | L22.1 | L44.1 | L92.0 | M05.0 | M31.8 | M35.1 |
| D69.01 | H61.0 | L12.11 | L22.2 | L44.2 | L93.0 | M05.2 | M32.0 | M35.2 |
| D69.02 | I89.8 | L12.2 | L30.42 | L51.0 | L93.1 | M06.3 | M32.9 | M35.3 |
| D69.8 | K12.0 | L12.21 | L40.11 | L51.1 | L93.2 | M08.2 | M33.0 | M35.4 |
| D86.3 | L10.0 | L12.3 | L43.0 | L51.2 | L95.1 | M15.1 | M33.1 | N48.1 |
| D86.31 | L10.1 | L13.0 | L43.1 | L52.1 | L95.8 | M30.0 | M34.1 | N48.11 |
| D86.32 | L10.2 | L13.1 | L43.3 | L52.2 | L95.9 | M30.3 | M34.8 | N48.12 |
| D86.33 | L10.21 | L13.9 | L43.8 | L52.3 | L98.2 | M31.3 | M34.81 | N48.2 |
| D86.34 | L10.4 | L21.11 | L43.81 | L57.01 | L98.3 | M31.4 | M34.9 | O26.4 |
| D86.8 | L10.8 | L22.0 | L43.9 | L57.5 | L98.8 | M31.6 | M35.0 | P00.8 |
| D89.1 | L12.0 |  |  |  |  |  |  |  |
| **Dermatoses due to microorganism** | | | | |  |  |  |  |
| A01.0 | A30.81 | A65. | A79.9 | B16.9 | B37.25 | B65.3 | H00.11 | L08.1 |
| A06.7 | A30.82 | A65.1 | A90.0 | B26.9 | B37.3 | B73.0 | I74.9 | L08.8 |
| A18.40 | A30.9 | A66.0 | B00.0 | B27.9 | B37.4 | B74.0 | J02.9 | L08.92 |
| A18.41 | A31.1 | A66.1 | B00.1 | B34.9 | B37.8 | B74.1 | L00.0 | L30.9 |
| A18.42 | A31.11 | A66.3 | B00.2 | B35.0 | B37.81 | B74.3 | L00.1 | L44.4 |
| A18.43 | A31.12 | A66.4 | B00.8 | B35.1 | B37.82 | B74.8 | L01.0 | L44.8 |
| A18.44 | A31.9 | A66.5 | B00.9 | B35.11 | B37.83 | B74.9 | L01.02 | L88.1 |
| A18.45 | A35.0 | A66.6 | B01.9 | B35.2 | B37.84 | B76.9 | L01.1 | L98.81 |
| A18.46 | A36.3 | A66.7 | B02.2 | B35.3 | B37.9 | B78.1 | L02.9 | L98.82 |
| A18.47 | A38.0 | A66.8 | B02.3 | B35.4 | B38.3 | B80.0 | L02.91 | N45.9 |
| A18.48 | A39.1 | A66.9 | B02.7 | B35.6 | B39.9 | B85.0 | L02.92 | N72.1 |
| A18.49 | A39.4 | A67.9 | B02.9 | B35.8 | B40.9 | B85.1 | L02.93 | N75.1 |
| A20.0 | A40.9 | A69.1 | B03.0 | B35.81 | B41.9 | B85.2 | L03.0 | N76.0 |
| A20.1 | A41.0 | A69.2 | B05.9 | B35.9 | B42.9 | B85.3 | L03.01 | N76.01 |
| A22.0 | A41.8 | A69.21 | B06.9 | B36.0 | B43.0 | B86.0 | L03.03 | N76.2 |
| A24.0 | A41.9 | A75.0 | B08.0 | B36.1 | B43.2 | B86.1 | L03.04 | N76.3 |
| A26.0 | A42.8 | A75.1 | B08.01 | B36.2 | B45.2 | B87.0 | L03.9 | N76.41 |
| A28.1 | A46.0 | A75.2 | B08.1 | B36.3 | B47.0 | B88.0 | L03.94 | N89.8 |
| A30.0 | A48.0 | A75.3 | B08.2 | B36.8 | B48.0 | B88.01 | L04.9 | P37.5 |
| A30.1 | A48.8 | A75.9 | B08.3 | B37.0 | B48.1 | B88.1 | L05.0 | P38.0 |
| A30.2 | A59.0 | A77.0 | B08.4 | B37.2 | B55.0 | B88.8 | L05.9 | T81.4 |
| A30.3 | A59.01 | A77.1 | B08.5 | B37.21 | B55.1 | B88.9 | L08.0 | T81.41 |
| A30.4 | A59.02 | A77.9 | B08.8 | B37.22 | B55.2 | B96.5 | L08.02 | T81.42 |
| A30.5 | A59.8 | A79.0 | B08.81 | B37.23 | B55.9 | H00.0 | L08.03 | T88.1 |
| A30.8 | A59.9 | A79.1 | B09.0 | B37.24 | B58.9 | H00.1 | L08.04 |  |
| **Malignant dermatoses** | | |  |  |  |  |  |  |
| B21.0 | C44.9 | C44.94 | C46.0 | C49.95 | C79.2 | C84.41 | C84.52 | C95.9 |
| C21.0 | C44.90 | C44.95 | C49.9 | C49.96 | C79.8 | C84.5 | C90.0 | D47.7 |
| C21.8 | C44.91 | C44.96 | C49.91 | C49.97 | C81.9 | C84.51 | C91.9 | D04 |
| C43.6 | C44.911 | C44.97 | C49.92 | C50.0 | C84.0 | C85.1 | C92.9 | D72.8 |
| C43.7 | C44.92 | C44.98 | C49.93 | C69.9 | C84.1 | C85.9 | C93.9 | D76.0 |
| C43.9 | C44.93 | C44.99 | C49.94 | C77.9 | C84.4 |  |  |  |
| **Hair and nail disorders** | | | |  |  |  |  |  |
| E70.32 | L60.2 | L60.38 | L60.86 | L65.11 | L66.1 | L67.8 | Q84.0 | Q84.22 |
| E70.33 | L60.3 | L60.4 | L60.9 | L65.2 | L66.2 | L67.81 | Q84.1 | Q84.23 |
| H30.8 | L60.30 | L60.5 | L63.0 | L65.8 | L66.3 | L67.82 | Q84.11 | Q84.3 |
| L01.01 | L60.31 | L60.8 | L63.2 | L65.81 | L66.8 | L67.83 | Q84.12 | Q84.31 |
| L01.03 | L60.32 | L60.81 | L63.9 | L65.82 | L66.81 | L67.9 | Q84.13 | Q84.4 |
| L08.81 | L60.33 | L60.82 | L64.9 | L65.83 | L66.9 | L67.91 | Q84.14 | Q84.5 |
| L21.02 | L60.34 | L60.83 | L65.0 | L65.9 | L67.0 | L68.0 | Q84.15 | Q84.6 |
| L58.11 | L60.35 | L60.84 | L65.01 | L65.91 | L67.1 | L68.1 | Q84.16 | Q84.61 |
| L60.0 | L60.36 | L60.85 | L65.1 | L65.92 | L67.11 | L68.9 | Q84.2 | Q84.62 |
| L60.1 | L60.37 |  |  |  |  |  |  |  |
| **Pigment disorders** | | |  |  |  |  |  |  |
| E70.3 | L81.01 | L81.21 | L81.411 | L81.44 | L81.6 | L81.63 | L81.72 | L81.81 |
| E70.31 | L81.1 | L81.3 | L81.42 | L81.49 | L81.61 | L81.7 | L81.8 | L81.9 |
| L81.0 | L81.2 | L81.4 | L81.43 | L81.5 | L81.62 | L81.71 |  |  |
