# Supplementary material for: Reorganising dermatology care: predictors of the substitution of secondary care with primary care
Source: BMC Health Serv Res. 2020 Jun 5;20:510. doi: 10.1186/s12913-020-05368-2 (PMC7275501; doi:10.1186/s12913-020-05368-2)
Supplement: Supplementary file 3 — Additional file 3. Comparison of patient categories. Comparison of patients and consultation characteristics of included and excluded patients. [file 12913_2020_5368_MOESM3_ESM.docx]

**Comparison of patient categories**

Table 1 Comparison of patients and consultation characteristics of included and excluded patients

|  | **Included patients**  **(N = 2,812)** | **Excluded patients**  **(N = 140)** | **p-values** |
| --- | --- | --- | --- |
| **Age in years (mean ± SD)** | 47.7 ± 20.9 | 48.3 ± 20.6 | 0.748 |
| **Gender – male % (N)** | 41.2 (1,159) | 37.9 (53) | 0.430 |
| **Number of consultations**  **(mean ± SD)** | 1.19 ± 0.4 | 1.10 ± 0.40 | 0.009* |
| **Specialist** |  |  | 0.002* |
| Specialist 1 % (N) | 53.6 (1,508) | 48.6 (68) |  |
| Specialist 2 % (N) | 25.6 (721) | 27.1 (38) |  |
| Specialist 3 % (N) | 11.1 (311) | 5.7 (8) |  |
| Other % (N) | 9.7 (272) | 18.6 (26) |  |
| **Diagnosis** |  |  | ≤0.001** |
| Naevi % (N) | 14.5 (407) | 2.1 (3) |  |
| Premalignant dermatosis % (N) | 9.4 (264) | 15.0 (21) |  |
| Benign tumours % (N) | 8.5 (238) | 2.1 (3) |  |
| Other eczema % (N) | 7.8 (219) | 14.3 (20) |  |
| Acneiform dermatoses % (N) | 6.1 (172) | 9.3 (13) |  |
| Inflammatory dermatoses % (N) | 5.7 (161) | 10.0 (14) |  |
| Dermatoses due to microorganism % (N) | 5.3 (149) | 6.4 (9) |  |
| Malignant dermatoses % (N) | 5.2 (146) | 1.4 (2) |  |
| Hair and nail disorders % (N) | 3.7 (103) | 0.7 (1) |  |
| Pigment disorders % (N) | 3.3 (94) | 7.9 (11) |  |
| Other % (N) | 23.2 (653) | 26.4 (37) |  |
| Unknown % (N) | 7.3 (206) | 4.3 (6) |  |

*SD = standard deviation
* P < 0.01; ** P* ≤ *0.001*
