# Supplementary material for: Reorganising dermatology care: predictors of the substitution of secondary care with primary care
Source: BMC Health Serv Res. 2020 Jun 5;20:510. doi: 10.1186/s12913-020-05368-2 (PMC7275501; doi:10.1186/s12913-020-05368-2)
Supplement: Supplementary file 4 — Additional file 4. Distribution of dermatological complaints. Comparison of the distribution of dermatological complaints in primary care, Primary Care Plus and secondary care. [file 12913_2020_5368_MOESM4_ESM.docx]

**Distribution of dermatological complaints**

Table 1 Comparison of the distribution of dermatological complaints in primary care, Primary Care Plus and secondary care

| **Diagnosis** | **Primary care (General Practitioner) %** | **Primary Care Plus  %** | **Secondary care (outpatient hospital care)**  **%** |
| --- | --- | --- | --- |
| Naevi | 3.0 | 14.5 | 9.0 |
| Premaligne dermatosis | - | 9.4 | 10.0 |
| Benign tumours | 9.0 | 8.5 | 7.0 |
| Other eczema * | 15.0 | 7.8 | 12.0 |
| Acneiform dermatoses | 2.0 | 6.1 | 6.0 |
| Inflammatory dermatoses | 18.0 | 5.7 | 4.0 |
| Dermatoses due to  microorganisms | - | 5.3 | - |
| Malignant dermatoses | 1.0 | 5.2 | 12.0 |
| Hair and nail disorders | - | 3.7 | - |
| Pigment disorders | - | 3.3 | - |
| Other | 52.0 | 23.2 | 40.0 |

** In primary care and secondary care, the category eczema was not further specified.*
